# Digital informed consent for urological surgery - randomized controlled study comparing multimedia-supported vs. traditional paper-based informed consent concerning satisfaction, anxiety, information gain and time efficiency

**DOI:** 10.1038/s41391-023-00737-4

**Published:** 2023-11-04

**Authors:** Maximilian Haack, Nikita D. Fischer, Lisa Frey, Peter Sparwasser, Robert Dotzauer, Gregor Duwe, Axel Haferkamp, Hendrik Borgmann

**Affiliations:** 1 Department of Urology and Pediatric Urology, University Medical Center, Mainz, Germany; 2 Department of Urology and Pediatric Urology, University Medical Center, Brandenburg an der Havel, Germany

**Keywords:** Outcomes research, Cancer screening

## Abstract

**Introduction:**

Due to a lack of time and staff, informed consent (IC) in clinical practice often lacks clarity, comprehensibility and scope of information. Digital media offer great potential to enhance IC. Aim of this study is to evaluate the effectiveness of multimedia-supported compared to traditional paper-based IC.

**Methods:**

In the randomized, controlled, three-arm DICon (Digital Informed Consent for urological surgery) study 70 patients with an indication for prostate biopsy were randomized 1:1:1 to receive traditional paper-based IC vs. multimedia-supported information before IC vs. multimedia-supported information during IC. Patient satisfaction, anxiety and information gain were measured by validated questionnaires 2 weeks and directly before the procedure and time efficiency was recorded. Statistical analysis was performed using Kruskal–Wallis and Dunn’s test (one-way ANOVA) and two-way ANOVA (with bonferroni post-test).

**Results:**

Multimedia information prior to the consultation saved 32.9% time compared to paper-based (5.3 min. vs. 9.5 min; *p* < 0.05) and 60.4% time compared to shared multimedia information (5.3 min. vs. 13.9 min.; *p* < 0.001), with no difference in satisfaction (62.6 vs. 62.7 vs. 68.6 of max. 80; *p* = 0.07), anxiety (8 vs. 8.1 vs. 7 of max. 16; *p* = 0.35), or information gain (6.5 vs. 5.7 vs. 6.7 of max. 10; *p* = 0.23). Results on satisfaction (56.6 vs. 62.6 vs. 66; *p* = 0.06), anxiety (7.2 vs. 7.2 vs. 6.8; *p* = 0.84), and information gain (7 vs. 6.4 vs. 5.9; *p* = 0.43) remained stable over time.

**Conclusions:**

Multimedia-supported IC prior to consultation provided improved time efficiency (33% gain) compared to traditional paper-based IC, with comparable satisfaction, anxiety and information gain. Multimedia-supported information materials should therefore be used more frequently in patient education.

## Introduction

Informed consent (IC) is mandatory before every surgical intervention and should inform the patient about expected consequences, risks, necessity, urgency, prospects of success and alternatives in a clear and comprehensive way [1–4]. Furthermore, detailed documentation of the consultation is needed and information material of IC should be handed out [1, 3]. Structured and comprehensive documentation improves patient care and reduces treatment errors [5]. However, studies have shown that 50% of physician’s working time consists of documentation and other desk tasks and only 27% on direct patient consultation [6–8]. Due to increasing documentation requirements, the high demands on IC become even more challenging. This disproportion is further exacerbated by rising patient numbers because of demographic change [9] and an aggravating shortfall of staff [10, 11]. Moreover, due to fast-tacked workflows in modern hospitals and multiple forms and questionnaires patients have to fill out, there often is not enough time or motivation to read the written IC-form completely and thoroughly [12]. In addition, many patients have problems understanding, because of language barriers. Furthermore, patient’s level of information about the respective intervention prior to the consultation can vary greatly and depends on many factors. Age, level of education, access to medical information and profession are just a few. But also, the affinity and compatibility to digital media can be a decisive limiting factor in that regard [13].

All the problems and obstacles listed above call for innovations in more comprehensive, descriptive, but also more time-effective educational alternatives. Digital media offer great potential in this respect and are being used more and more frequently [12, 14–17]. Because digital media can appeal to several senses, information can be better internalized [18, 19]. In addition, multimedia-assisted education can be more standardized through recorded videos or presentations, as these are always the same. Therefore, there is more time for individual questions and concerns. Moreover, different levels of experience and motivation of the clinician or a lack of time during the patient interview become less relevant factors for a comprehensive and satisfactory education [18].

Unfortunately, high acquisition and licensing costs of software programs as well as data protection concerns are still a major obstacle to digitalization [20, 21]. The aim of this study was to evaluate the effectiveness of an easily accessible, multimedia-supported patient education in comparison to a classical paper-based education, in terms of patient satisfaction, anxiety, disease knowledge and time efficiency.

## Patients and methods

### Study design and population

Between October 2021 and October 2022, a total of 70 patients indicated for prostate biopsy were included in this prospective, randomized, single-center study. Patients were divided into three groups. The first group represents the control group with a classic, paper-based information (PAPER). Here, each patient received the information forms in advance of the consultation and then discussed them with the physician during the consultation. The procedure and risks were illustrated only with the help of the printed material. Patients in the second group (MMprior) received a multimedia presentation specially prepared for prostate biopsy in addition to the printed information material before the consultation. The presentation was shown to the patient alone in a separate room. Afterwards, the multimedia presentation was discussed with the physician and questions or comments were clarified. The difference to the third group (MMtogether) was that the multimedia presentation was carried out by the physician within the framework of the consultation. In this way, questions or comments on specific topics of the procedure could be answered directly. The patient also received the printed information forms before the consultation, as was the case in all three groups. Outcome parameters were patient satisfaction, anxiety, information gain of about disease and procedure as well as consultation time. Randomization was performed using a list randomly generated by Microsoft® Excel® (Version 2308, Microsoft Corporation, Redmond, USA). According to this list, the patients were assigned to the appropriate group.

### Multimedia-supported information

The multimedia-supported information was realized by a Microsoft® PowerPoint® presentation (Version 2301, Microsoft Corporation, Redmond, USA) specially created for the prostate biopsy with information about anatomy, basic knowledge of prostate carcinoma, risks and side effects of the intervention, further procedure after the intervention and oncological prospects. Besides some illustrations and diagrams, the presentation included a short video (45 s), which illustrated the anatomy and procedure even further. The PowerPoint presentation is provided in Supplementary Appendix/Supplementary Material.

### Data acquisition

Subsequently to the consultation, the patient was given questionnaires for the evaluation of satisfaction, anxiety, as well as information gain. A second survey took place between 1–5 days before the planned intervention and was be carried out again with the same questionnaires that were used for the first interview. Thus, the sustainability of the information gain could be captured. To evaluate the time-efficiency of each group, the time of each consultation was recorded using a stopwatch. Patients who did not complete the first and second survey were excluded from the data analysis. This results in different sample numbers for the respective outcome parameters (satisfaction: *n* = 60; anxiety: *n* = 63; disease knowledge: *n* = 63). The recording of time was also inconclusive in a few cases, so that these were excluded. As a result, time recording was available for 62 patients.

### Questionnaires

Patient satisfaction was evaluated using a questionnaire (21 items, max. score 80) based on the standardized EORTC questionnaire “QLQ-IN-PATSAT32” for in-patient cancer care [22]. Here, a high score represents a high level of satisfaction. Anxiety was surveyed by the “Perceived Stress Scale” (PSS-4; 4 items, max. score 16), also known as the “Cohens-Scale” [23]. Unlike the satisfaction questionnaires, a high score in PSS-4 represents a high level of anxiety and distress. As far as the query of the information gain of disease knowledge is concerned, a multiple-choice test with 10 questions (max. score 10) was created.

### Statistical analysis

Statistical analysis was performed using Kruskal–Wallis and Dunn’s test (one-way ANOVA) and two-way ANOVA (with bonferroni post-test). All data were analyzed using GraphPad PRISM® 5 (Version 5.01, 2007, GraphPad Software Inc., Boston, USA). Statistical significance was defined as *p* < 0.05.

## Results

### Study population and consultation times

**Fig. 1:**
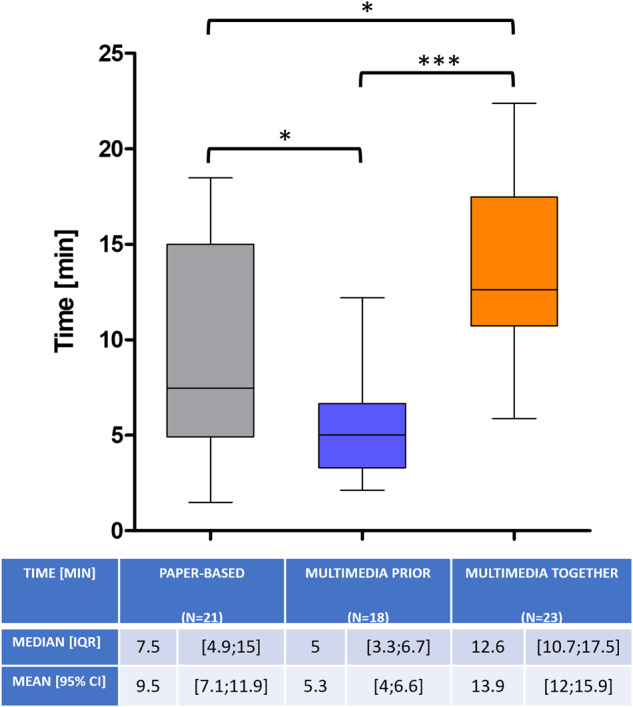
Consultation time [minutes]. Whiskers represent the 2.5–97.5 percentile. Presented below are Median [IQR] and Mean [95% CI]. Statistically significant was defined as *p* ≤ 0.05.

From all 70 patients surveyed, complete data could be collected at 60 for satisfaction, 63 for anxiety, 63 for information gain, and 62 for time. Mean patient age [with 95% Confidence Interval] was balanced between all three groups (68 [63.8;72], 64 [60;68.9] and 69 [64.9;73.1], *p* = 0.304). However, consultation times differed to each other (9.5 min [7.1;11.9], 5.3 min [4;6.6] and 13.9 min [12;15.9], *p* ≤ 0.0001) with a significant time saving in the MMprior-group of 32.9% compared to the PAPER-group (*p* < 0.05) and 60.4% to the MMtogether-group (*p* < 0.001) (Fig. 1). Dividing each of the groups into two age-categories (<65 and >65 years) there was still a significant time saving in the MMprior group in both age categories, compared to the MMtogether-group (<65: *p* = 0.0015; >65: *p* < 0.0001). Interestingly there was no significant difference in consultation time between the two age-categories in each group (PAPER: 9.3 min [5.1;13.5] vs. 9.6 min [6.2;13.1]; MMprior: 5.4 min [3.9;6.9] vs. 5.1 min [2.6;7.6]; MMtogether: 16 min [12.4;19.6] vs. 12.6 min [10.4;14.9]; *p* = 0.33).

### Satisfaction

**Fig. 2:**
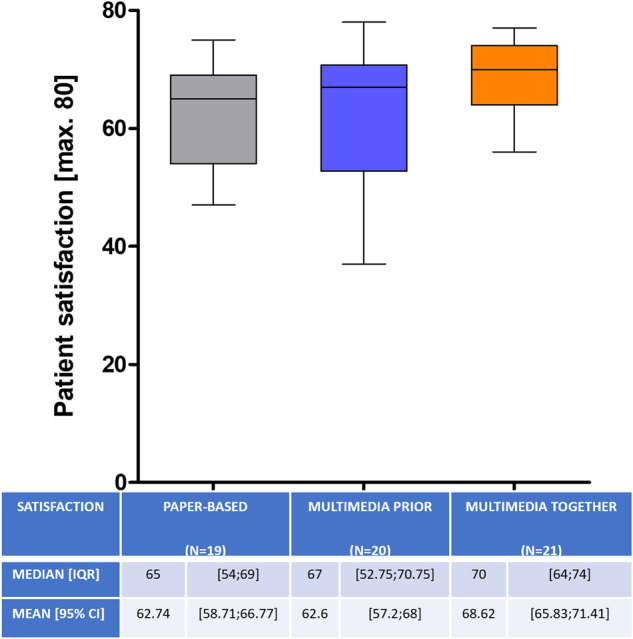
Patient satisfaction with a maximum score of 80. Whiskers represent the 2.5–97.5 percentile. Presented below are Median [IQR] and Mean [95% CI].

Mean patient satisfaction at first interview did not differ significantly between the three groups (62.6 vs. 62.7 vs. 68.6 of max. 80; *p* = 0.07) (Fig. 2). There was no significant difference in the second survey either (56.6 vs. 62.6 vs. 66; *p* = 0.06). Comparing the first to second interview there was no significant decrease in satisfaction in all three groups (*p* = 0.48).

### Anxiety

**Fig. 3:**
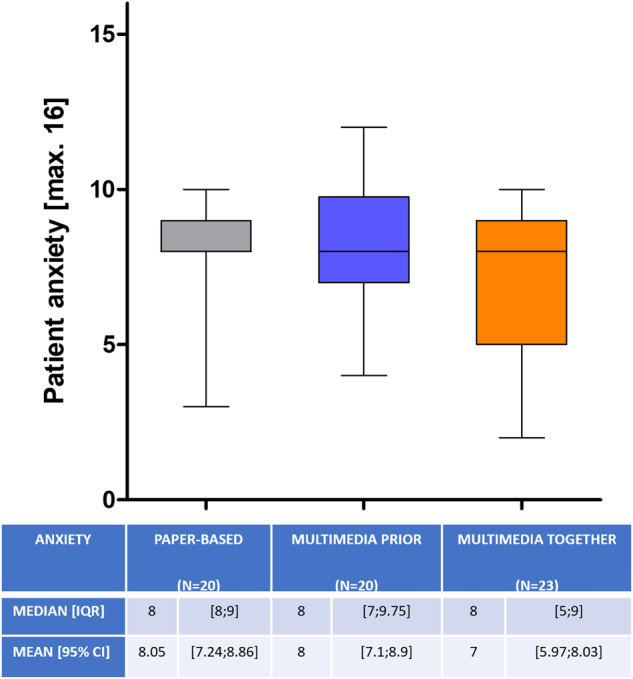
Patient anxiety with a maximum score of 16. Whiskers represent the 2.5–97.5 percentile. Presented below are Median [IQR] and Mean [95% CI].

Looking at patient’s anxiety, multimedia-assisted information did not show to be inferior regarding mean score at first (8 vs. 8.1 vs. 7 of max. 16; *p* = 0.35) (Fig. 3) or second survey (7.2 vs. 7.2 vs. 6.8; *p* = 0.84). The small difference between the first and second interview was also not statistically significant (*p* = 0.72).

### Disease knowledge

**Fig. 4:**
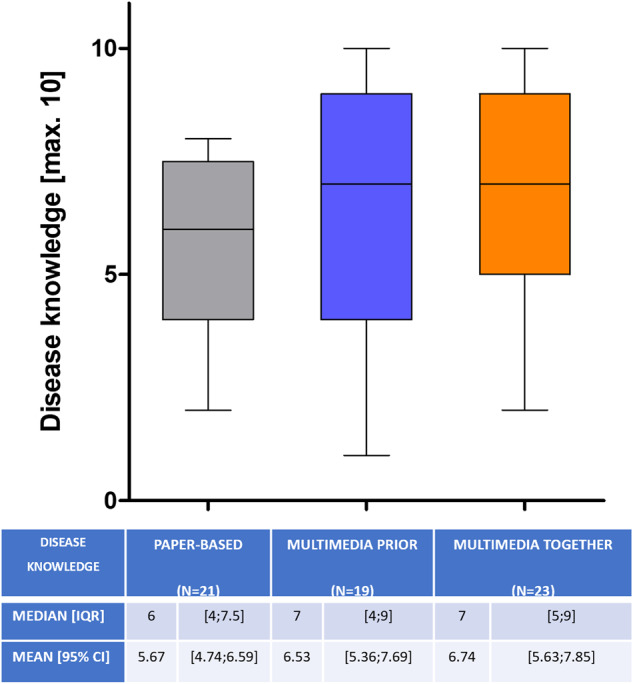
Disease knowledge with a maximum score of 10. Whiskers represent the 2.5–97.5 percentile. Presented below are Median [IQR] and Mean [95% CI].

In terms of disease knowledge, patients who received multimedia information did not perform worse than the traditional paper-based group (6.5 vs. 5.7 vs. 6.7 of max. 10; *p* = 0.23) (Fig. 4). The information could be imparted in an equally sustainable way throughout all three groups, because the different scores at the second survey (7 vs. 6.4 vs. 5.9; *p* = 0.43) were not statistically significant compared to the first interview (*p* = 0.33). Dividing each group into two age-categories (<65 vs. >65 years) the difference was not statistically significant in the first survey (PAPER: 6.1 [4.5;7.7] vs. 5.3 [4.1;6.6]; MMprior: 7.3 [6.1;8.4] vs. 6.5 [4.7;8.3]; MMtogether: 7.5 [5.9;9.1] vs. 6.2 [4.5;7.8]; *p* > 0.05 in all three comparisons, overall *p* = 0.104). Even at second survey the two age-categories scored statistically indifferent in the disease knowledge test (PAPER: 7.8 [6.4;9.2] vs. 5.4 [2.7;8.1]; MMprior: 6.6 [3.8;9.3] vs. 6.7 [4.7;8.8]; MMtogether: 6.3 [4.3;8.4] vs. 5.7 [4.1;7.2]; *p* > 0.05 in all three comparisons, overall *p* = 0.213).

## Discussion

The process of IC is subject to high requirements, so that a patient can make a differentiated decision on a surgical intervention. However studies have shown, that patients with an oncological disease in particular, often decide on treatment options based on personal or someone else’s experience as well as the physician’s recommendation [24–26]. However, these influences can be biased because of personal interests or fear of the same bad experiences and complications someone else’s had [27]. Therefore, a comprehensive, detailed and empathic education from the physician is indispensable. Unfortunately, due to staff shortages, high patient volumes and documentation obligations, the modern hospital routine no longer offers sufficient space to meet these requirements [6, 7, 9, 10]. Digital media may not cover the empathic part of the consultation, but can greatly support with information transfer and vividness [14, 28]. Especially considering, that waiting times could be used more effectively with the use of digital educational materials. Digital tools for patient education have already been developed and have shown their effectiveness [14, 17]. However, high acquisition costs and license fees often mean that modern digital media are being cut back [29, 30]. These challenges gave us the reason to evaluate an easily accessible, cost-efficient, multimedia-supported patient education tool for patients indicated for prostate biopsy.

As digitalization plays a central role in societies worldwide, the use of digital media in elderly populations (>65 years) has increased considerably over the last ten years [31]. However age still shows to be a limiting factor in the use of digital media in general [32, 33]. Therefore, we had concerns about older patients in our study population having problems coping with the multimedia assisted IC, especially when it was shown to them alone. Thus, we created the MMtogether-group, where the physician could guide the patient through the multimedia-assisted presentation. Interestingly we could not detect a significant difference in test performance between the two age-categories (<65 and >65 years). Also, there was no difference in satisfaction (Fig. 2), anxiety (Fig. 3) and disease knowledge (Fig. 4) in comparison to the MMtogether-group. Thus, we can state that widely used multimedia software (Microsoft® PowerPoint® presentation) specially prepared for prostate biopsy is compatible with elderly patients and can safely be shown in a sole setting. Moreover, it is very cost-effective, as clinics often already have basic Software tools such as Microsoft® Office. Before trying to implement complex, time-consuming and expensive multimedia tools for IC, we suggest using easily accessible and cost-effective software, which can be created and distributed quickly for many procedures and treatments. Putting this in relation to the time saved by using multimedia-assisted IC (Fig. 1), it shows a considerable benefit of simple digital media. Our data show a time saving of 32,9% (Fig. 1) in consultation time while maintaining the same level of satisfaction, anxiety and disease knowledge. Therefore, our data can verify the effectiveness of digital media in terms of timesaving compared to other studies [14, 18, 34, 35]. Looking at the shortage of staff and increasing patient numbers [9, 10] the demonstrated time-effectiveness of multimedia-assisted IC could become even more relevant in the near future. With increasing access to digital media and internet, multimedia information could be made available to patients online or in the waiting room so that they receive clinic-specific specialist information in a comprehensible and vivid manner in advance of the appointment.

In summary, our study provides significant data that support the time-effectiveness of multimedia-assisted IC tools, without compromising patient satisfaction, anxiety and information gain on disease knowledge. Our data further show that widely used digital media is compatible with elderly patients and can safely be shown in a sole setting. Through further technological developments and advancing digitalization in societies worldwide, the challenge of high demands on patient education, documentation obligations, increasing patient numbers and lack of medical staff could be managed with the little time available.

Our study also has some limitations. The study population was relatively small and patient education was carried out by several physicians. Unfortunately, we did not perform an analysis of statistical power prior to this study. Thus, a too small sample number could be the reason for the lack of significant differences in satisfaction, anxiety and information gain. Furthermore, the multimedia-assisted presentation and questionnaires were only available in German language, which could hinder comprehensibility. In addition, we only tested the multimedia-assisted IC tool on male patients with indication for prostate biopsy. It is unclear how the outcome parameters would be affected in the context of a more complex operation or balanced gender ratios. To evaluate the gender imbalance, a follow-up study is already being conducted on patients indicated for transurethral resection of bladder tumors. Moreover, there was a problem of questionnaire completeness at second survey, because of scheduling difficulties due to the COVID-19 pandemic. These patients were excluded from data analysis.

## Conclusion

For patient consent, multimedia information material prior to consultation promotes time efficiency, without compromising patient satisfaction, anxiety and information gain to purely paper-based education, even in elderly patient populations. Multimedia information materials offer solutions to an increasing divergence of time and resources in healthcare and should therefore be used more frequently.

## Supplementary information

Supplementary material

## Data Availability

The datasets generated during and/or analyzed during the current study are available from the corresponding author on reasonable request.
