# Supplementary material for: Digital informed consent for urological surgery - randomized controlled study comparing multimedia-supported vs. traditional paper-based informed consent concerning satisfaction, anxiety, information gain and time efficiency
Source: Prostate Cancer Prostatic Dis. 2023 Nov 4;27(4):715–9. doi: 10.1038/s41391-023-00737-4 (PMC11543590; doi:10.1038/s41391-023-00737-4)
Supplement: Supplementary file 1 — Supplementary material [file 41391_2023_737_MOESM1_ESM.pptx]

## Slide 1
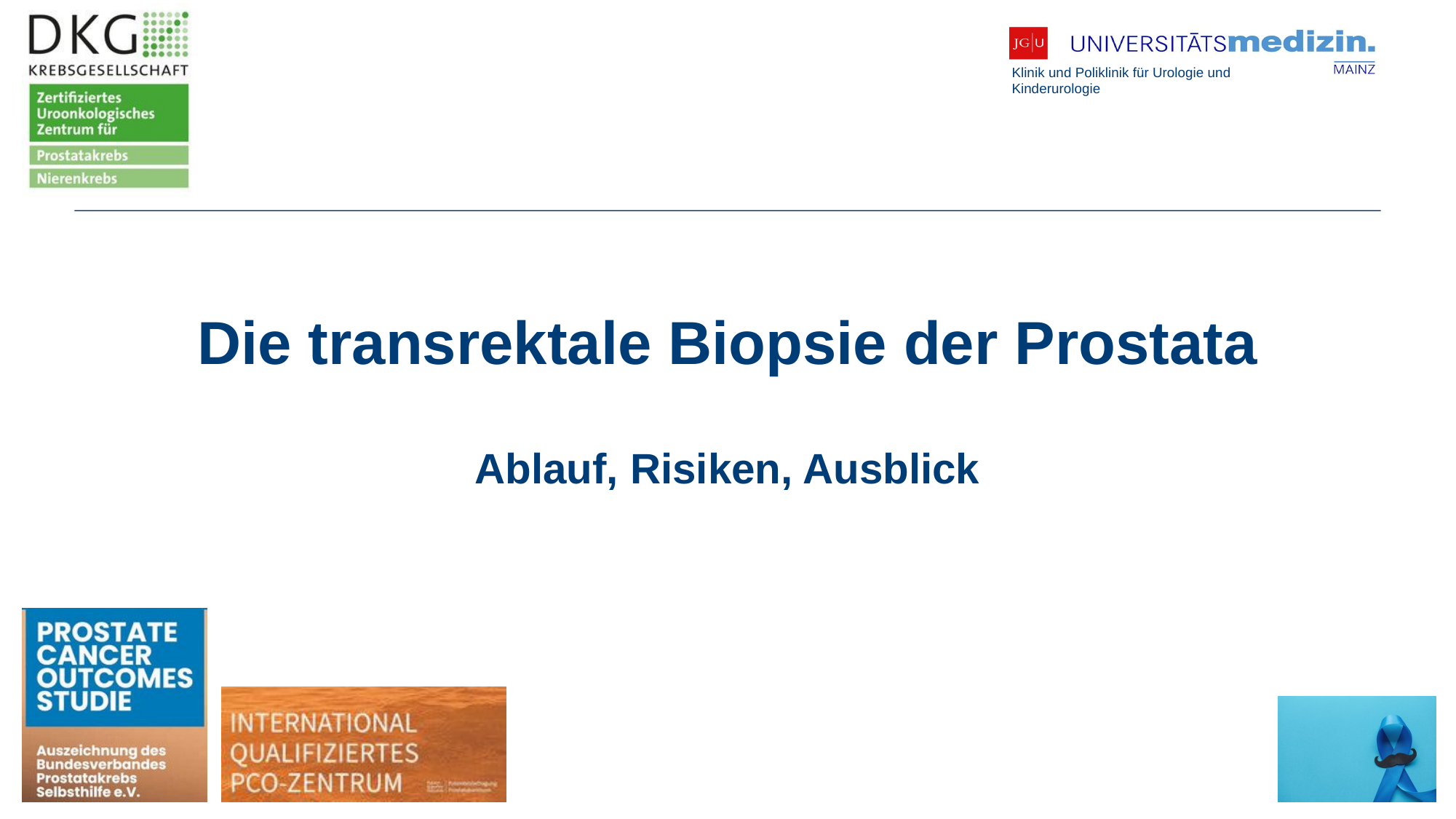

# Die transrektale Biopsie der Prostata
Ablauf, Risiken, Ausblick

## Slide 2
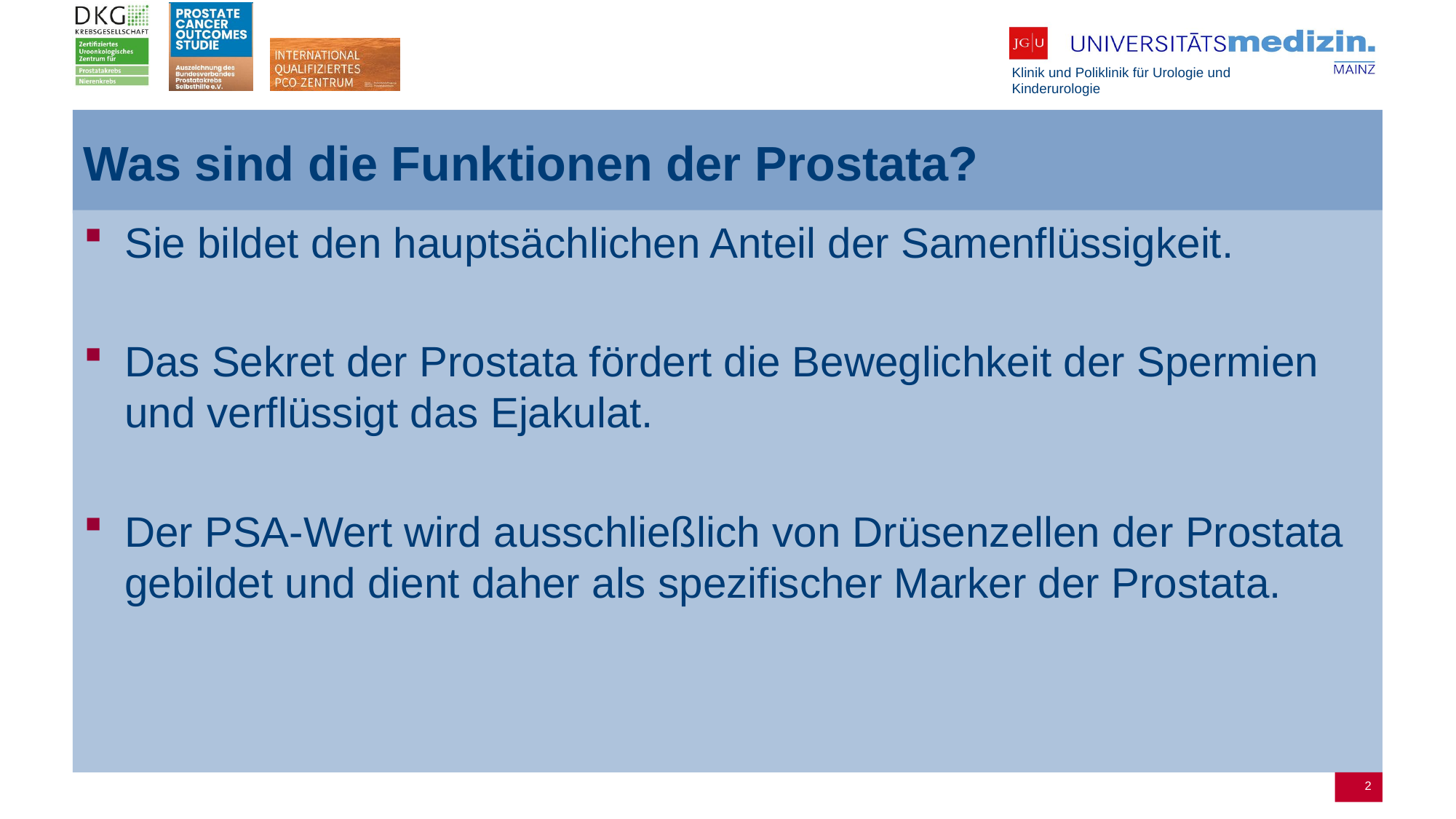

# Was sind die Funktionen der Prostata?
Sie bildet den hauptsächlichen Anteil der Samenflüssigkeit.
Das Sekret der Prostata fördert die Beweglichkeit der Spermien und verflüssigt das Ejakulat.
Der PSA-Wert wird ausschließlich von Drüsenzellen der Prostata gebildet und dient daher als spezifischer Marker der Prostata.
2

## Slide 3
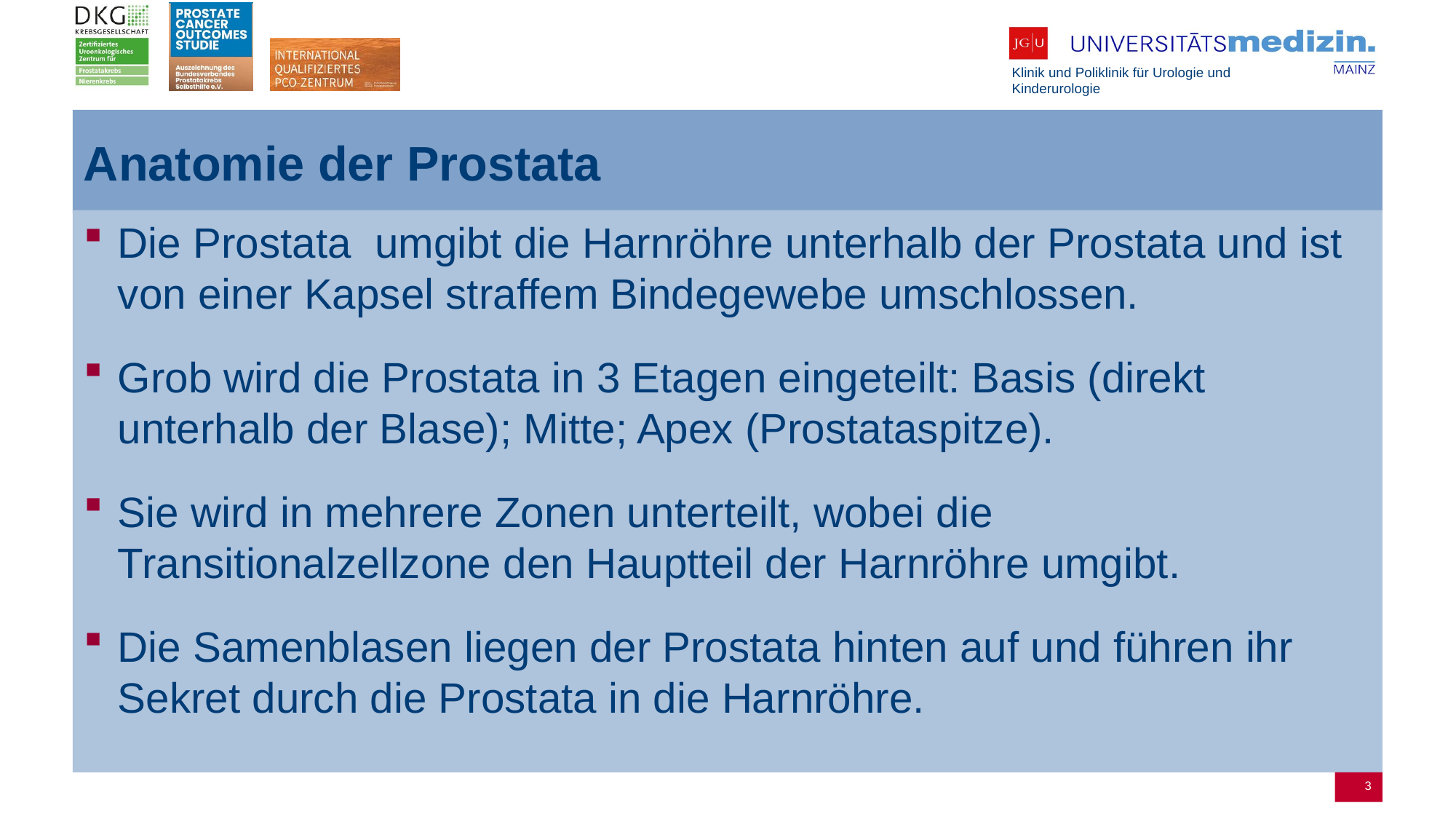

# Anatomie der Prostata
Die Prostata  umgibt die Harnröhre unterhalb der Prostata und ist von einer Kapsel straffem Bindegewebe umschlossen.
Grob wird die Prostata in 3 Etagen eingeteilt: Basis (direkt unterhalb der Blase); Mitte; Apex (Prostataspitze).
Sie wird in mehrere Zonen unterteilt, wobei die Transitionalzellzone den Hauptteil der Harnröhre umgibt.
Die Samenblasen liegen der Prostata hinten auf und führen ihr Sekret durch die Prostata in die Harnröhre.
3

## Slide 4
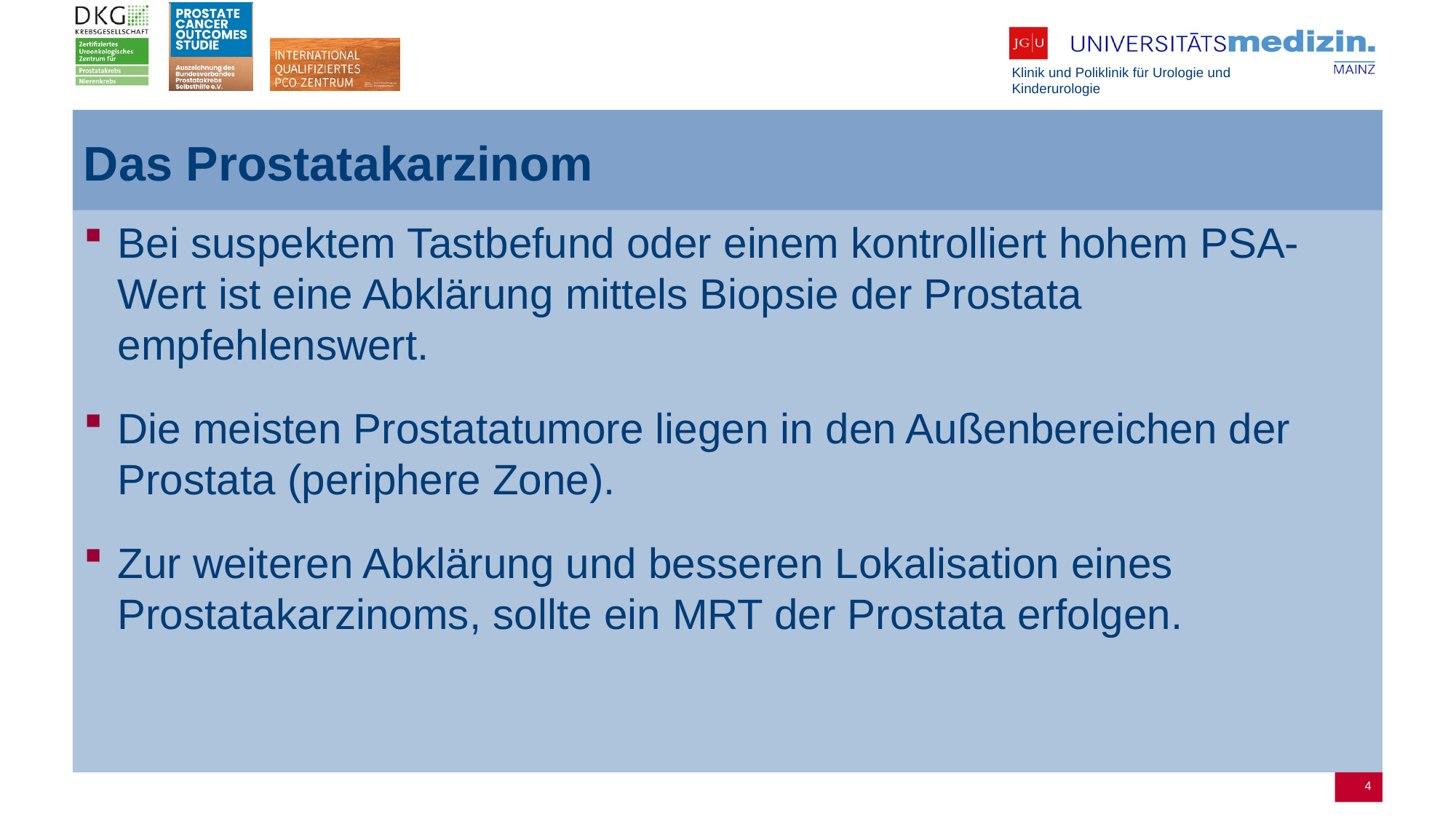

# Das Prostatakarzinom
Bei suspektem Tastbefund oder einem kontrolliert hohem PSA-Wert ist eine Abklärung mittels Biopsie der Prostata empfehlenswert.
Die meisten Prostatatumore liegen in den Außenbereichen der Prostata (periphere Zone).
Zur weiteren Abklärung und besseren Lokalisation eines Prostatakarzinoms, sollte ein MRT der Prostata erfolgen.
4

## Slide 5
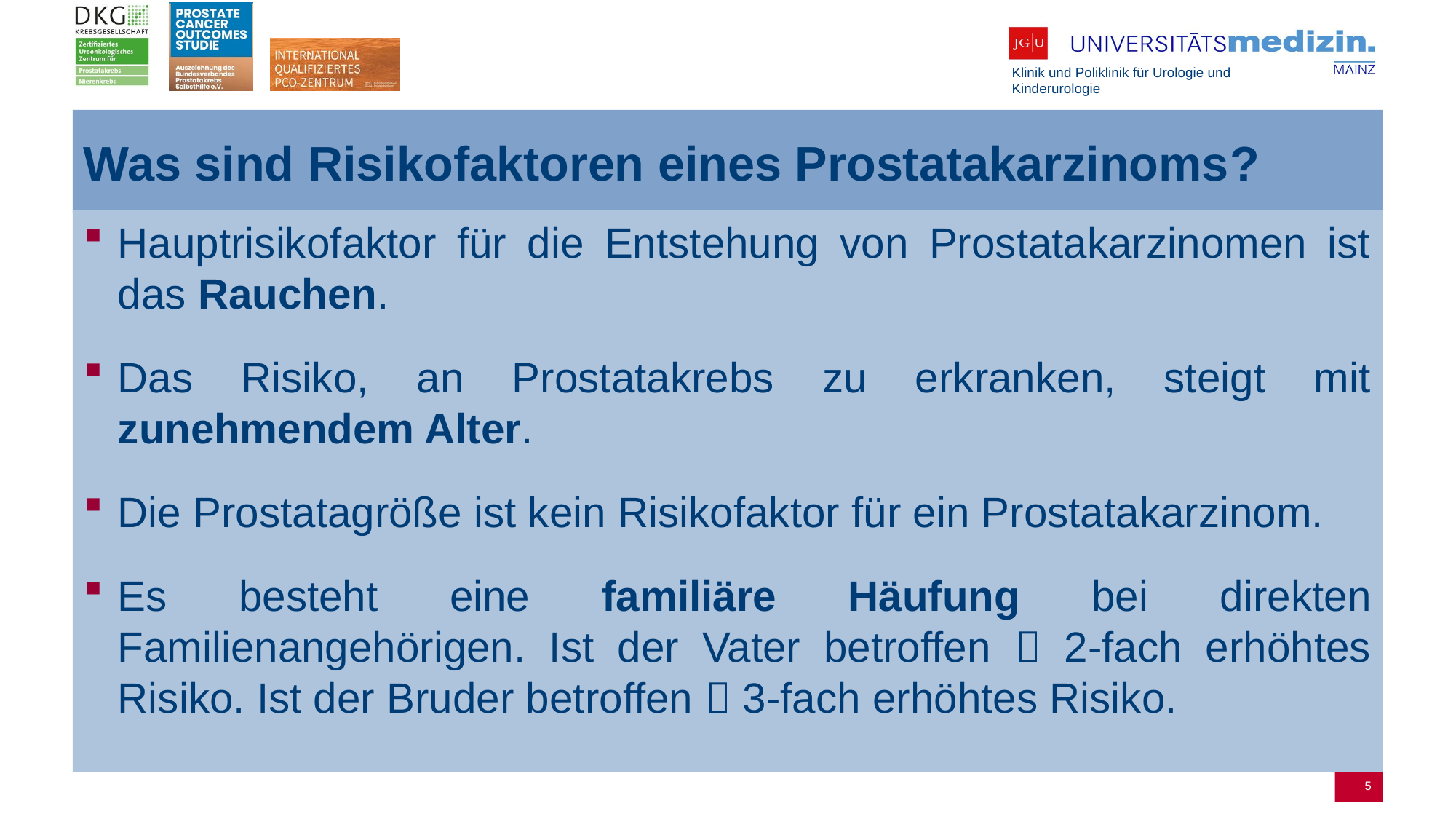

# Was sind Risikofaktoren eines Prostatakarzinoms?
Hauptrisikofaktor für die Entstehung von Prostatakarzinomen ist das Rauchen.
Das Risiko, an Prostatakrebs zu erkranken, steigt mit zunehmendem Alter.
Die Prostatagröße ist kein Risikofaktor für ein Prostatakarzinom.
Es besteht eine familiäre Häufung bei direkten Familienangehörigen. Ist der Vater betroffen  2-fach erhöhtes Risiko. Ist der Bruder betroffen  3-fach erhöhtes Risiko.
5

## Slide 6
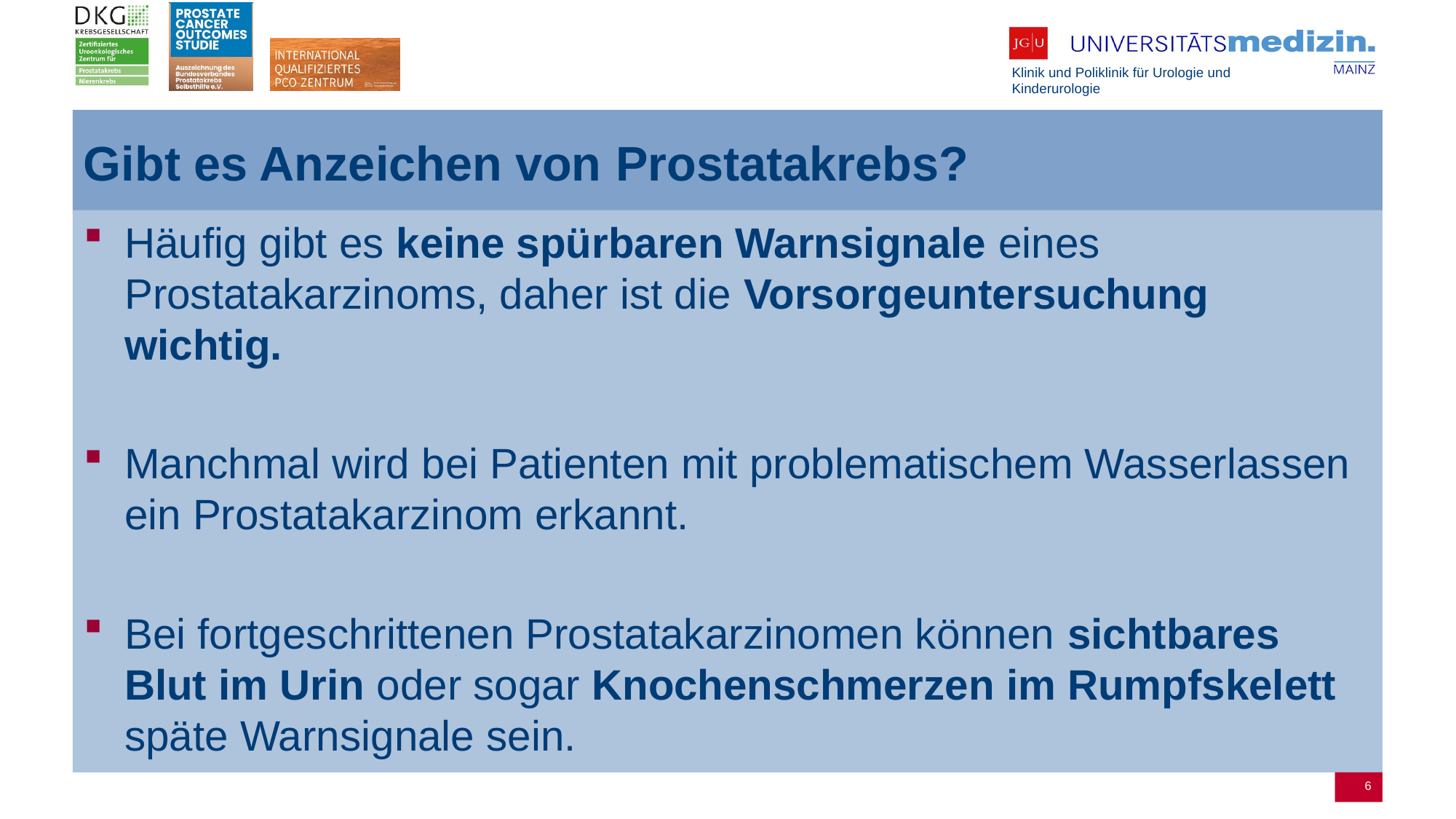

# Gibt es Anzeichen von Prostatakrebs?
Häufig gibt es keine spürbaren Warnsignale eines Prostatakarzinoms, daher ist die Vorsorgeuntersuchung wichtig.
Manchmal wird bei Patienten mit problematischem Wasserlassen ein Prostatakarzinom erkannt.
Bei fortgeschrittenen Prostatakarzinomen können sichtbares Blut im Urin oder sogar Knochenschmerzen im Rumpfskelett späte Warnsignale sein.
6

## Slide 7
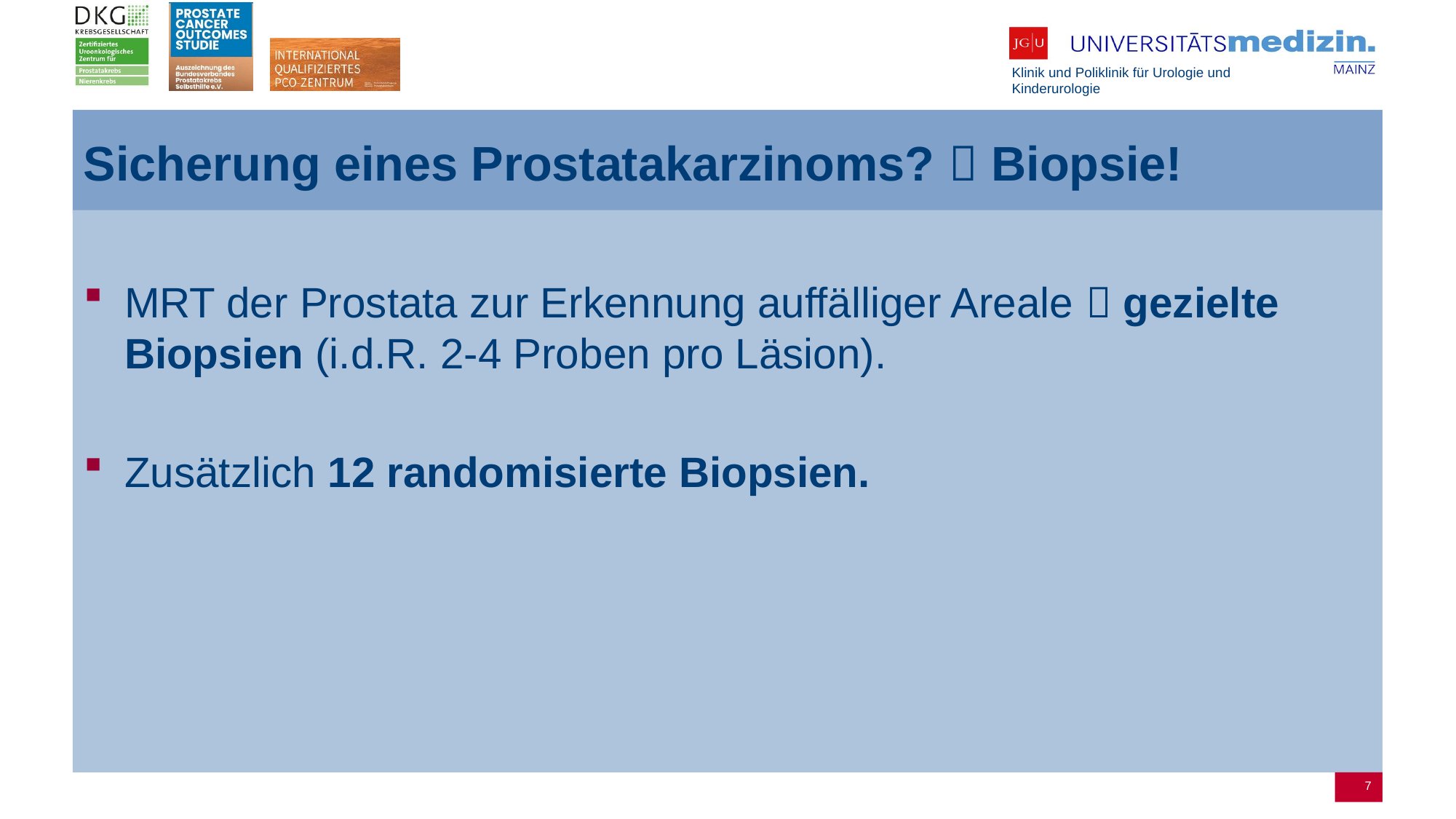

# Sicherung eines Prostatakarzinoms?  Biopsie!
MRT der Prostata zur Erkennung auffälliger Areale  gezielte Biopsien (i.d.R. 2-4 Proben pro Läsion).
Zusätzlich 12 randomisierte Biopsien.
7

## Slide 8
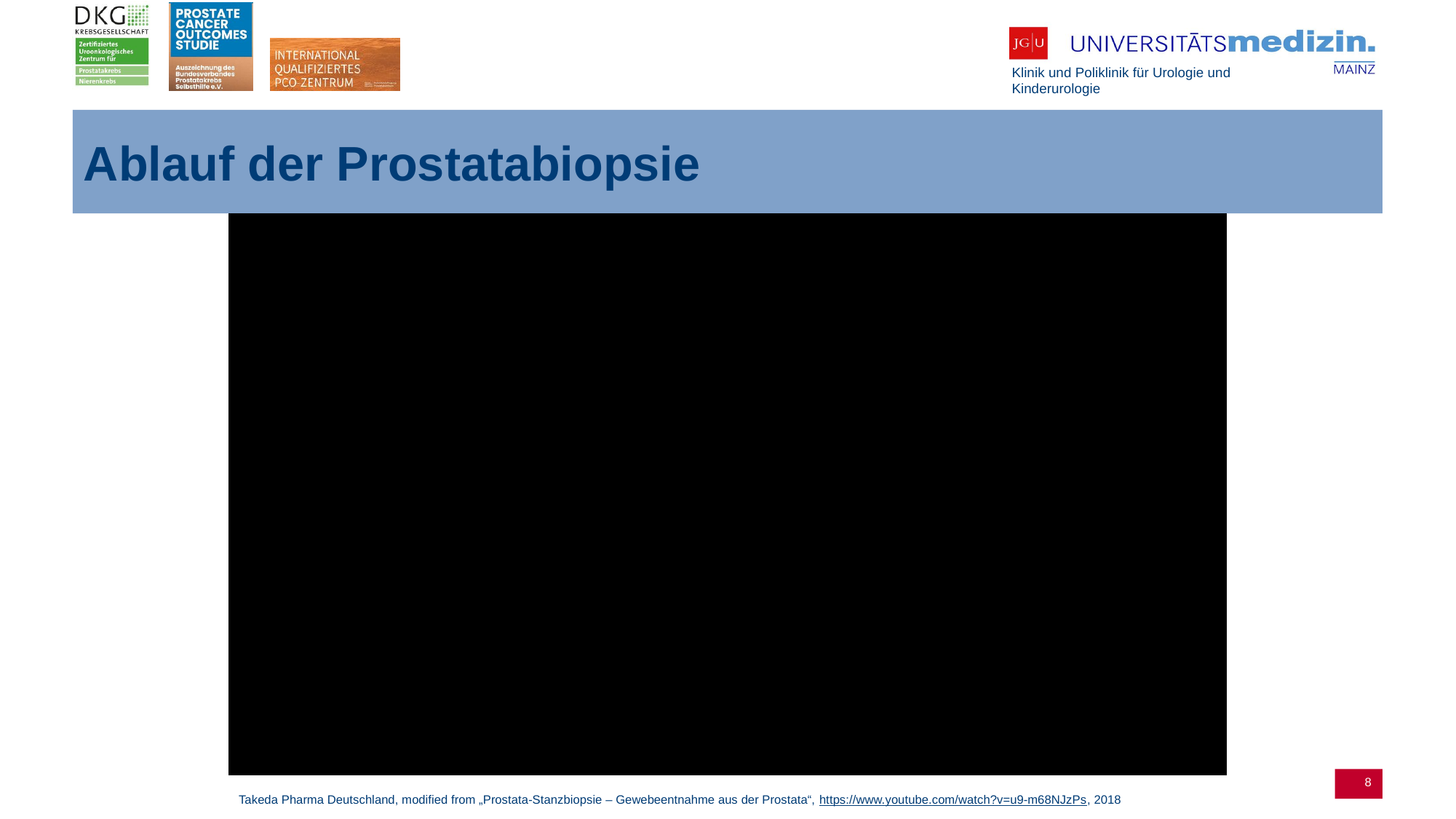

# Ablauf der Prostatabiopsie
8
Takeda Pharma Deutschland, modified from „Prostata-Stanzbiopsie – Gewebeentnahme aus der Prostata“, https://www.youtube.com/watch?v=u9-m68NJzPs, 2018

## Slide 9
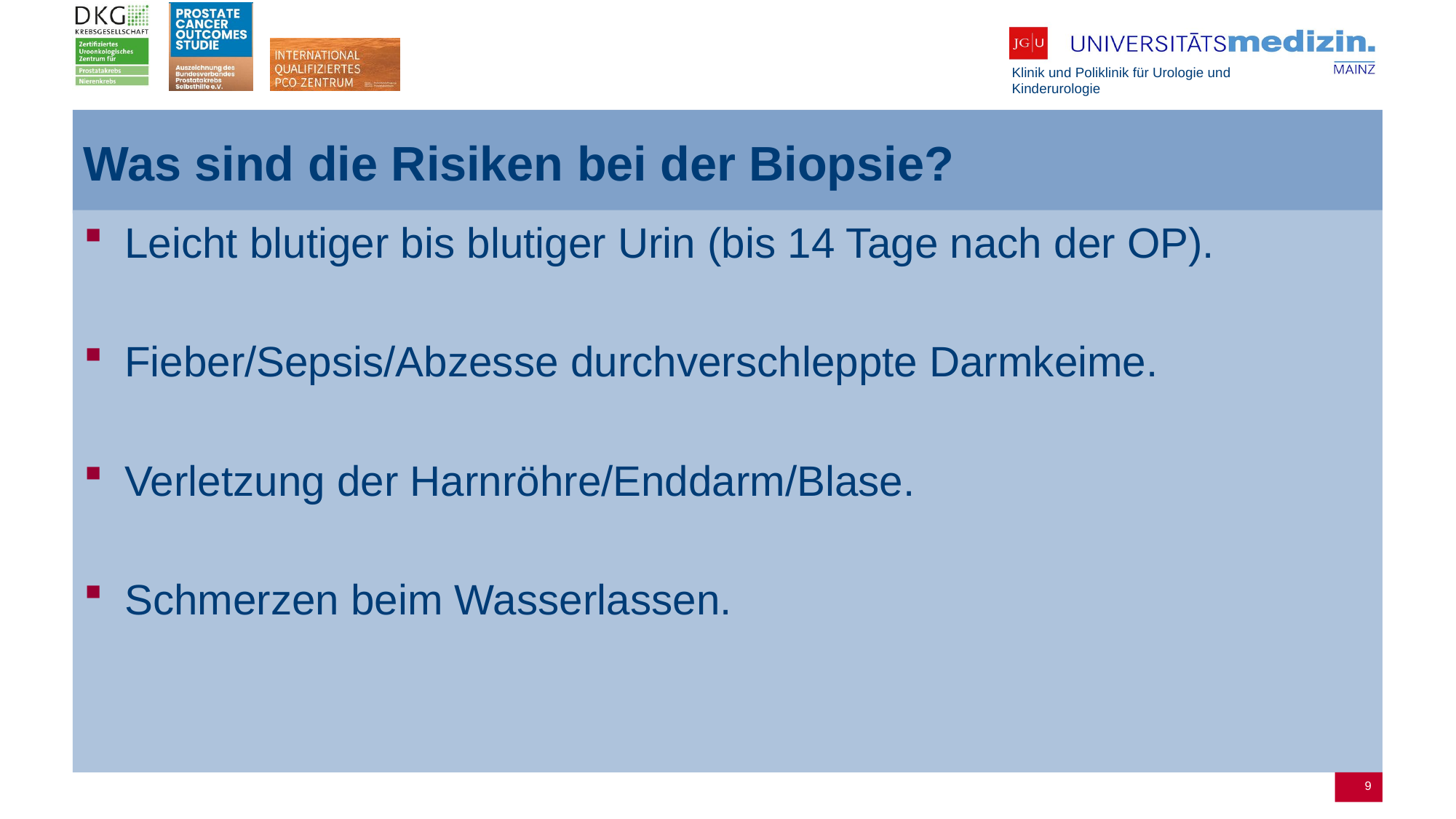

# Was sind die Risiken bei der Biopsie?
Leicht blutiger bis blutiger Urin (bis 14 Tage nach der OP).
Fieber/Sepsis/Abzesse durchverschleppte Darmkeime.
Verletzung der Harnröhre/Enddarm/Blase.
Schmerzen beim Wasserlassen.
9

## Slide 10
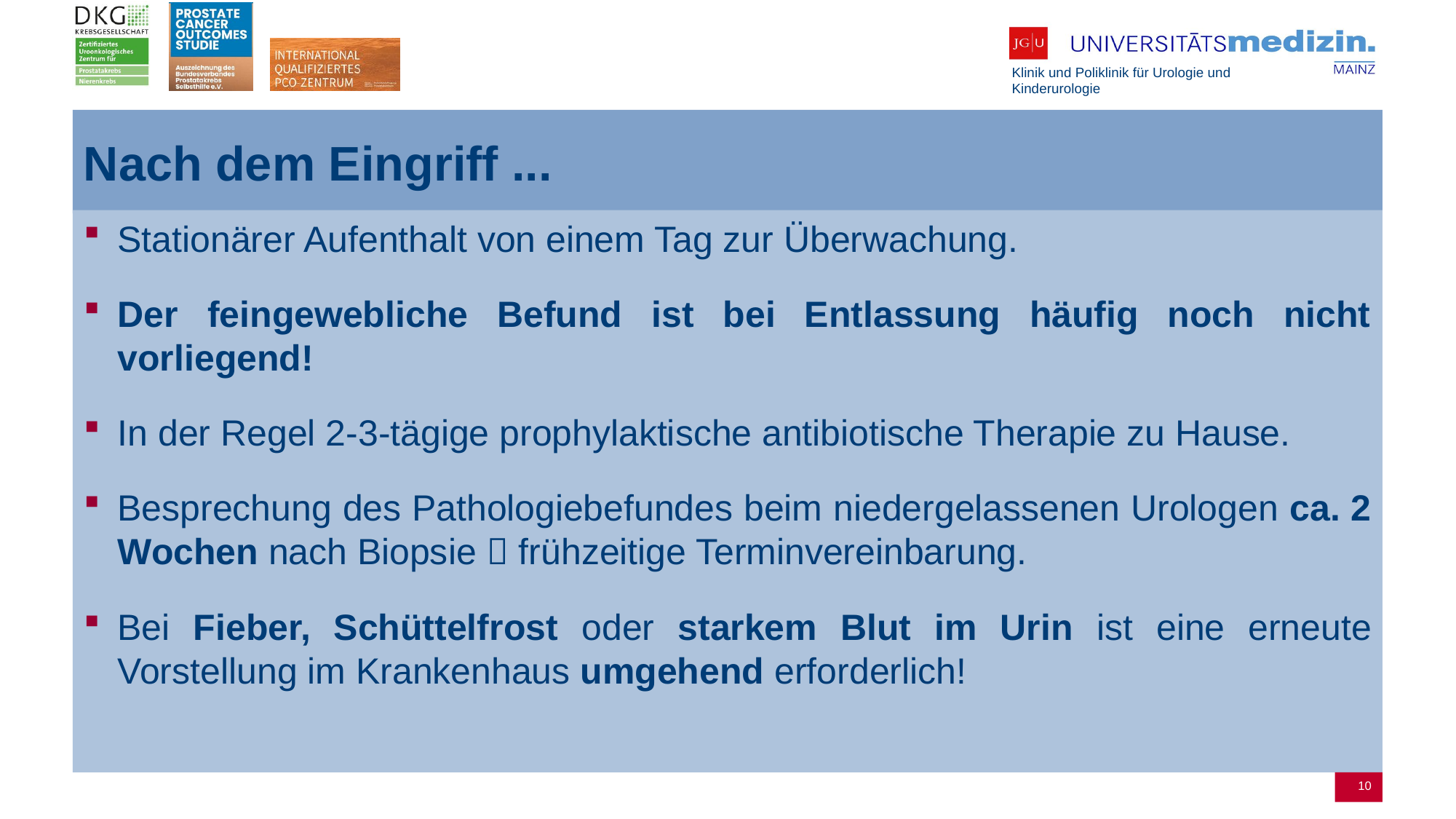

# Nach dem Eingriff ...
Stationärer Aufenthalt von einem Tag zur Überwachung.
Der feingewebliche Befund ist bei Entlassung häufig noch nicht vorliegend!
In der Regel 2-3-tägige prophylaktische antibiotische Therapie zu Hause.
Besprechung des Pathologiebefundes beim niedergelassenen Urologen ca. 2 Wochen nach Biopsie  frühzeitige Terminvereinbarung.
Bei Fieber, Schüttelfrost oder starkem Blut im Urin ist eine erneute Vorstellung im Krankenhaus umgehend erforderlich!
10

## Slide 11
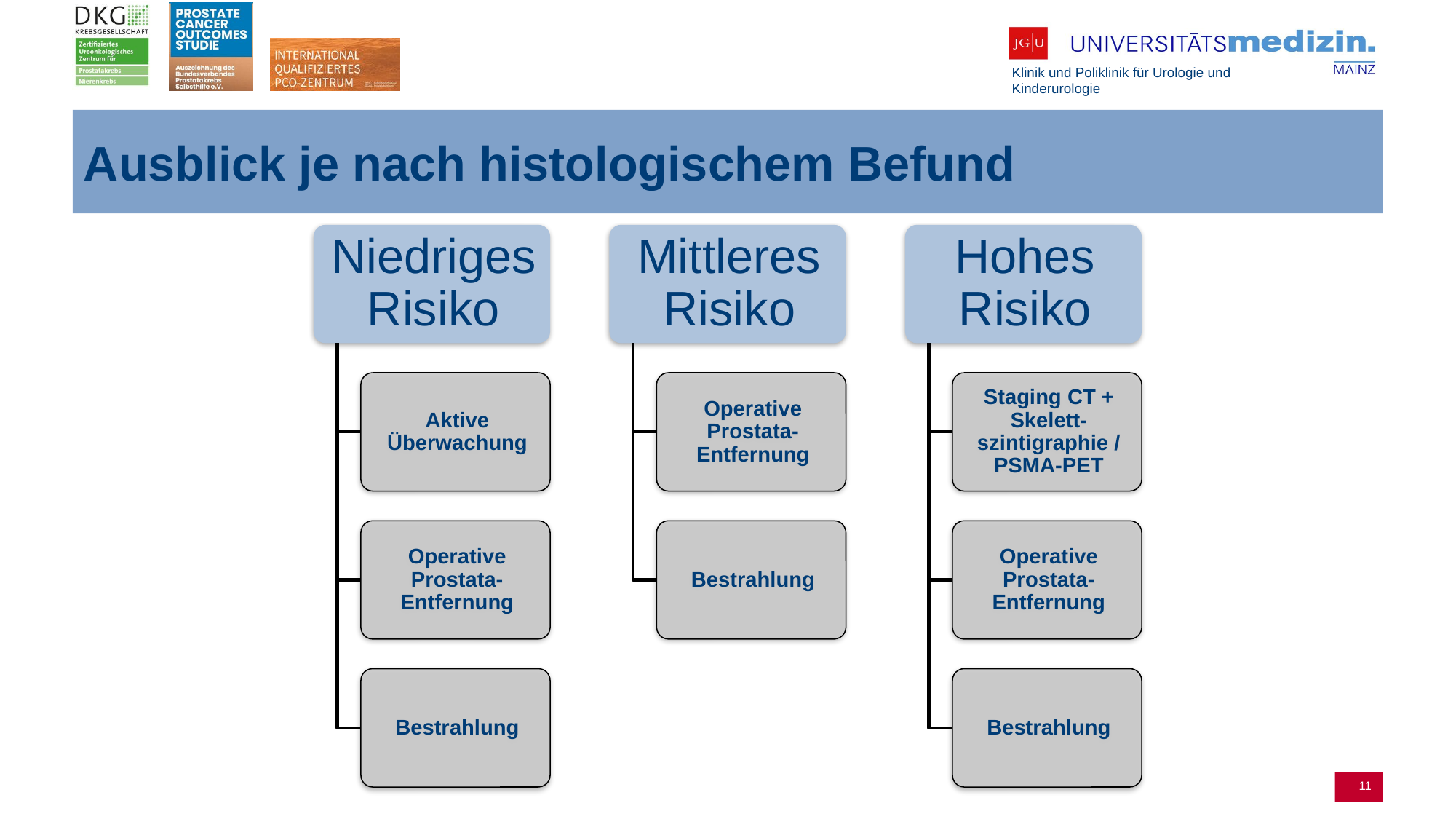

# Ausblick je nach histologischem Befund
11

## Slide 12
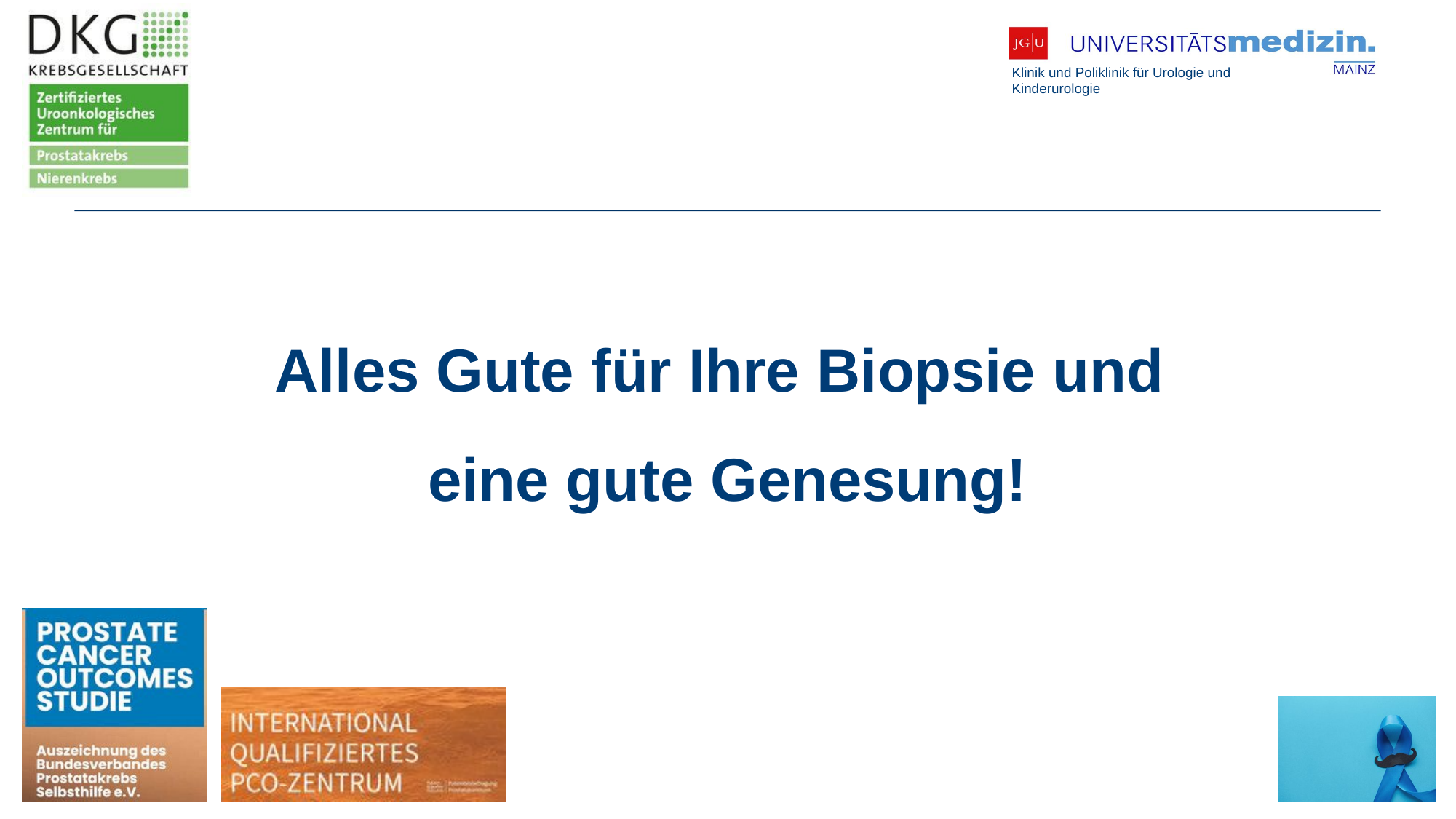

# Alles Gute für Ihre Biopsie und eine gute Genesung!
